# Lower Cardiovascular Stress during Resistance Training Performed with Inter-Repetition Rests in Elderly Coronary Patients

**DOI:** 10.3390/medicina56060264

**Published:** 2020-05-28

**Authors:** Olga Ribeiro-Torres, Arilson Fernandes M. de Sousa, Eliseo Iglesias-Soler, Maelán Fontes-Villalba, Hassane Zouhal, François Carré, Carl Foster, Daniel Boullosa

**Affiliations:** 1 School of Health and Medicine, Catholic University of Brasilia, Brasilia 71966-700, Brazil; olgartorres@gmail.com; 2 Physical Education, UNICEPLAC, Brasilia 72445-020, Brazil; arilsonf@hotmail.com; 3 Department of Physical Education and Sports, University of A Coruna, 15179 Bastiagueiro, Spain; eliseo.iglesias.soler@udc.es; 4 Faculty of Medicine, Lund University, 20502 Malmö, Sweden; maelan.fontes_villalba@med.lu.se; 5 Department of Sport Sciences, University of Rennes 2, 35043 Rennes, France; hassane.zouhal@univ-rennes2.fr; 6 Sports Medicine Department, University of Rennes 1, 35000 Rennes, France; francois.carre@univ-rennes1.fr; 7 Department of Exercise and Sport Science, University of Wisconsin-La Crosse, La Crosse, WI 54601, USA; cfoster@uwlax.edu; 8 INISA, Federal University of Mato Grosso do Sul, Campo Grande 79070-900, Brazil; 9 College of Healthcare Sciences, James Cook University, Townsville 4811, Australia

**Keywords:** cardiovascular stress, resistance exercise, resistance training, cardiac rehabilitation, set configuration

## Abstract

*Background and Objectives:* Hemodynamic stress during resistance training is often a reason why this training method is not used in cardiac patients. A lifting protocol that imposes rests between repetitions (IRRT) may provide less hemodynamic stress compared to traditional resistance training (TT). The aim of this study was to verify differences between set configurations on hemodynamic stress responses in resistance training. *Materials and Methods:* We compared hemodynamic (heart rate (HR), systolic blood pressure (SBP), and rate pressure product (RPP)) responses assessed with the auscultatory method in elderly (age = 75.3 ± 7.3 years) coronary male patients who were participating in a cardiac rehabilitation program allocated to either TT or IRRT with the same load (kg) and total number of repetitions (24) in the bilateral leg extension exercise. *Results:* IRRT resulted in significant lower values than TT for RPP at repetitions 8 (*p* = 0.024; G = 0.329; 95% CI: 0.061, 0.598) and 16 (*p* = 0.014; G = 0.483; 95% CI: 0.112, 0.854). *Conclusions:* IRRT appears to be a viable method of reducing the hemodynamic response (i.e., RPP) to resistance training and, thus, may contribute to the safety of cardiac rehabilitation programs. Further studies with more cardiac patients and other measurement techniques should be conducted to confirm these important findings.

## 1. Introduction

Resistance training (RT) has been traditionally considered less suitable for cardiac patients than aerobic training. The excessive pressure response observed in classical studies [1,2] was the main reason behind this conception. However, during the last years, a body of evidence has confirmed the appropriateness and effectiveness of this exercise mode, whether isolated or in combination with aerobic exercise, for improving body composition, muscular strength, aerobic capacity, and quality of life for these patients [3,4,5,6]. While some guidelines [7] still recommend the use of low-to-moderate intensities during RT for cardiac patients, more recent evidence suggests the use of dynamic RT exercises with high loads (>70% repetition maximum (RM)) and a reduced number of repetitions, as this approach results in reduced cardiovascular stress, namely heart rate (HR), cardiac output (CO), and blood pressure (BP) responses, while favoring greater strength improvements in an efficient manner [8,9,10,11,12].

The traditional approach for organizing RT session is to complete single or various sets with a determined number of repetitions and the targeted intensity in each exercise. More recently, a different set configuration called cluster or inter-repetition rest training (IRRT), which consists of including rest intervals after each single repetition, has been recommended and has been demonstrated to be very effective for maintaining mechanical performance (e.g., bar velocity and power) during RT sessions with less fatigue than traditional set configurations [13,14]. Previously, Iglesias-Soler et al. [15] showed in athletes that the IRRT in a squat exercise session elicited lower mean systolic BP (SBP) and rate pressure product (RPP) than a traditional set configuration (TT). More recently, Mayo et al. [16] found more SBP peaks but a lower HR and RPP in the recumbent leg press exercise with the IRRT when compared with TT in healthy, young individuals. While differences between studies regarding subjects’ characteristics, hemodynamic challenges associated to different exercises and loads, and measurement techniques used could further complicate the understanding of these previous evidence, it could be suggested that the IRRT is superior for cardiac patients given the lower cardiovascular stress expected during RT sessions with this set configuration.

Therefore, the objective of the current investigation was to describe and compare the changes in HR, SBP, and RPP during a RT session with TT and IRRT configurations in a sample of elderly coronary patients undergoing a cardiac rehabilitation program. Based on previous studies, our hypothesis was that mean SBP and RPP during the IRRT session would be lower than during the TT session.

## 2. Materials and Methods

### 2.1. Patients

Fifteen elderly (≥65 years old), stable coronary male patients with a minimum of one year of experience in RT (3 days a week) who regularly participated in a cardiac rehabilitation program were invited to participate in this study. All were informed about the procedures and the risks associated with the protocols and provided written informed consent. Inclusion criteria included completing all testing sessions, not using a pacemaker, no evidence of ischemia or arrhythmias, not presenting discomfort the days of evaluation, and maintaining medications prescribed during the study. One patient used a pacemaker, and another did not complete the evaluations. Thus, a final sample of 13 coronary male patients was included in this study. Their characteristics are included in Table 1. The local ethics committee (protocol number 79719317.6.0000.0029) approved this study in accordance with the tenets of the Declaration of Helsinki on 13 December 2017.

Patients presented different diagnosis before participation in the rehabilitation program including: hypertension (8), coronary insufficiency (2), acute myocardial infarction (5), cerebrovascular accident (1), myocardial revascularization surgery (2), dyslipidemia (5), diabetes mellitus type II (1), and coronary angioplasty (4). During the time of the study, the patients were taking beta-blockers (6), statins (8), cholesterol lowering drugs (5), antidiabetics (8), and antihypertensives (10).

### 2.2. Resistance Training Sessions

On a first day, the patients were evaluated for body composition and completed the physical activity readiness questionnaire (PAR-Q) [17] and the international physical activity questionnaire (IPAQ) [18]. Subsequently, after a brief warm up, they completed an 8-RM test in the bilateral leg extension machine with a progressive testing protocol following standard procedures [19]. The patients completed the exercises between 6:00 and 16:00 h and repeated the same time schedule in all testing sessions to avoid the influences of circadian rhythms on results.

Forty-eight hours after the first evaluation, the patients performed one of the experimental sessions in randomized order: TT, which consisted of 3 sets of 8 repetitions with the load (kg) determined in the 8-RM test of the first day with a passive recovery between sets of 3 min, and IRRT, which consisted of 1 set with the same load (kg) and number of repetitions (24) but with the total recovery time divided between each repetition (i.e., 15 s of rest between repetitions). Both protocols were performed with the maximal intended velocity and with a normal respiration avoiding the Valsalva maneuver. In a third day, the patients completed the other experimental session. Experimental sessions were separated by at least 48 h. During these sessions, HR was monitored with a HR monitor (RS800, Polar Electro Oy, Finland) and SBP was recorded by means of the auscultatory method by an experienced clinician before and after the 8th, 16th, and 24th repetitions and 10 min after the last repetition completed. Of note, given the short time interval for BP measurements, the cuff was inflated by ~10 s before the start of the recovery interval to ensure enough time for recording SBP in both conditions [20]. Subsequently, RPP was calculated for the same time points.

### 2.3. Statistical Analysis

Descriptive values are reported as mean ± SD. Normality assumption was verified by using the Shapiro–Wilk test. In order to contrast the evolution of HR, SBP, and RPP throughout the sessions, a two-way repeated measures ANOVA was performed. The factors considered for each ANOVA were session (i.e., IRRT and TT) and time (i.e., baseline; 8th, 16th, and 24th repetition; and 10 min post-session). When a significant session × time interaction was detected, pairwise comparisons were performed using paired *t*-tests with Bonferroni’s correction. Effect sizes for the pairwise comparisons are reported as Hedge’s g (*G*) with their corresponding 95% confidence intervals (CI). Additionally, a post hoc power analysis and a sensitivity analysis were performed. The statistical power for the interaction of the 2 × 5 repeated measures ANOVA, with a sample size of 13, a correlation among repeated measures of 0.7, and a medium effect size (f = 0.25) is 0.81. Additionally, the sensitivity of the tests for an alpha level of 0.05 and a power of 0.90 was sufficient for detecting a medium effect size (f = 0.28) for the interaction. All the analyses were carried out by using the statistical package SPSS version 20.0 (SPSS, IBM, Armonk, NY, USA) and the G Power software (version 3.1.9.2, Heinrich Heine University, Düsseldorf, Germany). The level of statistical significance was set at 0.05.

## 3. Results

**Figure 1:**
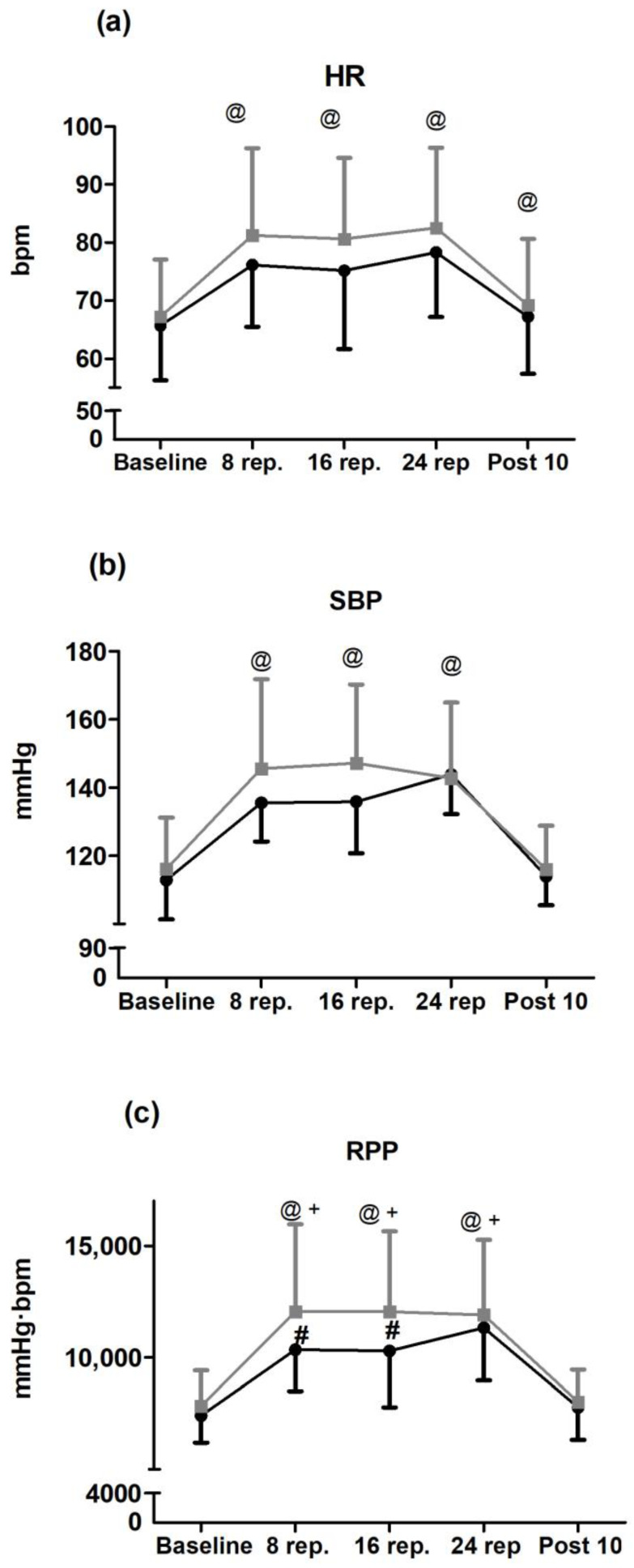
Hemodynamic responses during inter-repetition rest training (IRRT; black line) and traditional training (TT; grey line) sessions: (**a**) HR = heart rate; (**b**) SBP = systolic blood pressure; (**c**) RPP = rate pressure product. For HR and SBP, @ = *p* < 0.05 when comparing the pooled data from both sessions to baseline (i.e., main effect of time: *p* < 0.001 for both HR and SBP). For RPP, @ = *p* < 0.05 when compared to baseline during TT; + = *p* < 0.05 when compared to baseline during IRRT; and # = *p* < 0.05 when comparing TT vs. IRRT.

The results of ANOVA for HR, SBP, and RPP are show in Figure 1. Session × time interaction was only significant for RPP. Post hoc pairwise comparisons showed significant differences between conditions for repetitions 8 (*p* = 0.024; G = 0.329; 95% CI: 0.061, 0.598) and 16 (*p* = 0.014; G = 0.483; 95% CI: 0.112, 0.854).

## 4. Discussion

The results of the current study confirm our hypothesis of a smaller hemodynamic (i.e., RPP) stress during IRRT while performing bilateral leg extension exercise compared to TT with the same load (kg) and total number of repetitions. Specifically, there were significant time × session interactions only for RPP after the 8th and 16th repetitions. To the best of our knowledge, this is the first evidence in the literature reporting a reduced hemodynamic load during IRRT in a clinical population which agrees with previous reports with young healthy individuals [15,16]. These findings are of importance as a strategy for reducing cardiovascular risk during RT in these patients.

This study is not without limitations. First, recordings of SBP were with the auscultatory method and after lifting loads. Therefore, it would be expected that our SBP recordings were ~31% lower in both conditions than those measured with direct methods during lifting [21]. Future studies should be conducted with continuous recording of hemodynamic responses with other measurement techniques during the whole training session to verify potential differences during peak BP responses between set configurations [16]. Second, mechanical performances and perceptual fatigue were not recorded. Future studies should evaluate these parameters for confirming the superiority of IRRT regarding these outcomes in this population as in previous literature [14]. Lastly, the reduced sample size is a limitation that warrants further research with more cardiac patients of both sexes to verify the potential differences in HR and BP responses between different set structures. Meanwhile, the inclusion of elderly cardiac patients familiarized with the current protocols, under medical supervision, and with maintenance of their medication intakes are the major strengths of the current investigation providing excellent ecologic validity. Future studies are also needed to verify the safety and efficiency of this method in the long term and if the lowered cardiovascular stress is followed by post-exercise hypotension.

## 5. Conclusions

In summary, this study suggests that a cluster set configuration may induce a lower cardiovascular stress (i.e., RPP) in elderly coronary male patients when compared to a matched workload (kg × number of repetitions) with a traditional set configuration.

## Figures and Tables

**Table 1:** Sample’s characteristics (n = 13): The data are reported as mean ± SD.

Age (years)	75.4 ± 7.3
Height (m)	1.7 ± 0.1
Body Mass (kg)	72.2 ± 6.1
BMI (kg·m^−2^)	25.6 ± 1.8
Waist Circumference (cm)	92.4 ± 5.2
Hip Circumference (cm)	96.1 ± 4.8
Waist-to-Hip Ratio	0.9 ± 0.1
METs (MET-min·week^−1^)	2098 ± 1732
TV watching (min·week^−1^)	1084 ± 842
Basal HR (bpm)	69 ± 9
Basal SBP (mmHg)	113 ± 11
Basal DBP (mmHg)	70 ± 11
8-RM (kg)	40.46 ± 5.97

BMI = body mass index; MET = metabolic equivalent; HR = heart rate; SBP = systolic blood pressure; RPP = rate pressure product; RM = repetition maximum.
